# Water Softening in Hospitals

**Published:** 1909-03-06

**Authors:** 


### WATER SOFTENING IN HOSPITALS.

The advantages which follow the possession of an efficient supply of soft water in operating theatres seem hardly as yet to be realised in the surgical world; but the economical aspects of water-softening appliances are without doubt becoming more widely appreciated by those responsible for the construction and rebuilding of hospitals. The installation of a reliable water softener should affect to an appreciable degree the economy of a hospital or any institution with large laundry requirements. The saving on the soap account amounts to a considerable sum between one year's end and another, while the beneficial effect upon boilers and hot-water apparatus is shown by a material reduction in the expenses of repairs and renewals. We have previously described at length the excellent water softener which bears the name of Bruun-Lowener. Messrs. Lassen and Hjort, of 52 Queen Victoria Street, London, E.C., who are the English agents for this apparatus, will be pleased to give information and advice on the subject of water softening free of charge to our readers. Among recent hospital installations carried out by this firm may be mentioned those at University College Hospital, London; Holloway Sanatorium, Virginia Water; and the Woolwich Union Infirmary, Plumstead.

Welbank's Boilerettes, which formed the subject of very favourable criticism in our columns some time ago, deserve the attention of those medical men and hospital authorities who wish to know of a saucepan which is free from the defects of the ordinary utensil, and is therefore suited for the preparation of food for invalids and convalescents. In our opinion this cooking apparatus bears out the claims which are made on its behalf.

Nisbet's Medical Directory for 1909. (London: James Nisbet and Company, Limited. Price 7s. 6d. net; to subscribers, 4s. 2d. net, post free.)

The second edition of "Nisbet's Medical Directory." will increase the reputation of this new venture in medical reference books, and establish its position for the future. The present issue shows many small improvements and additions, which without making it any less compact add greatly to its completeness and utility. It is stated in the preface that 1,000 new entries have been made, but we cannot find that the bulk and weight of the volume have thereby been increased appreciably. The main features of this handy and inexpensive directory must no doubt be familiar to the majority of medical men, but we may repeat that the list of registered practitioners is arranged in the first part of the volume in alphabetical order without reference to the kingdoms in which they reside, and that the information concerning each medical man, which is reduced on a definite plan to the smallest proportions, commences with the qualification first obtained. The Directory is thus very convenient for rapid reference, and is a useful addition to the small handbooks on a busy practitioner's writing desk.

